# Diffusely impaired myocardial perfusion in patients with suspected coronary artery disease: Clinical utility of relative perfusion

**DOI:** 10.1111/cpf.70096

**Published:** 2026-09-29

**Authors:** Niklas Vartiainen, Antti‐Pekka E. Rissanen, Suvi Hartikainen, Svantte Halttunen, Aleksi J. Leikas, Elina Kariniemi, Eero Strengell, Paavo‐Ilari Kuikka, Juha E. K. Hartikainen, Tomi P. Laitinen

**Affiliations:** ^1^ Department of Clinical Physiology and Nuclear Medicine Kuopio University Hospital Kuopio Finland; ^2^ Helsinki Sports and Exercise Medicine Clinic (HULA), Foundation for Sports and Exercise Medicine Helsinki Finland; ^3^ Sports and Exercise Medicine, Faculty of Medicine University of Helsinki Helsinki Finland; ^4^ Department of Medicine Kuopio University Hospital Kuopio Finland; ^5^ Department of Clinical Physiology and Nuclear Medicine North Karelia Central Hospital Joensuu Finland; ^6^ A.I. Virtanen Institute for Molecular Sciences University of Eastern Finland Kuopio Finland; ^7^ Heart Center, Kuopio University Hospital Kuopio Finland; ^8^ Gene Therapy Unit, Kuopio University Hospital Kuopio Finland; ^9^ Department of Clinical Radiology Kuopio University Hospital Kuopio Finland; ^10^ Department of Clinical Physiology and Nuclear Medicine Mikkeli Central Hospital Mikkeli Finland; ^11^ Institute of Clinical Medicine University of Eastern Finland Kuopio Finland

**Keywords:** coronary artery disease, microvascular dysfunction, myocardial blood flow, myocardial perfusion, relative myocardial flow reserve, relative myocardial perfusion

## Abstract

**Background:**

In patients with suspected coronary artery disease (CAD), diffusely impaired hyperaemic myocardial blood flow (MBF) may indicate a multivessel epicardial CAD but also impaired coronary microcirculation. We assessed whether relative hyperaemic MBF—that is, the ratio of the lowest to the highest regional hyperaemic MBF—predicts obstructive CAD in patients with diffusely impaired hyperaemic MBF.

**Methods:**

We studied 874 patients who underwent coronary computed tomography angiography and [^15^O]H_2_O myocardial perfusion imaging with positron emission tomography at Kuopio University Hospital, Finland, in 2017–2023. The final analyses included 64 patients who had diffusely impaired hyperaemic MBF and were subsequently referred to invasive coronary angiography (ICA).

**Results:**

In ICA, 34/64 (53%) patients had obstructive CAD. Compared to patients with non‐obstructive CAD, those with obstructive CAD had lower relative hyperaemic MBF: 66% (95% CI 62–69%) versus 52% (95% CI 47–57%), respectively (*p* < 0.001). When relative hyperaemic MBF was used to predict the presence of obstructive CAD in ICA, the receiver operating characteristic analysis showed an area under the curve of 0.778 (95% CI 0.666–0.891). In binary logistic regression analysis, relative hyperaemic MBF independently predicted obstructive CAD (OR = 0.92 [95% CI 0.86–0.97], *p* = 0.004).

**Conclusion:**

Obstructive CAD is more likely in patients with lower relative hyperaemic MBF. Consequently, relative hyperaemic MBF may have clinical utility in patients with suspected CAD and diffusely impaired hyperaemic MBF.

## 1 INTRODUCTION

Coronary artery disease (CAD) is one of the leading causes of death worldwide (Safiri et al., 2022). Traditionally, the diagnosis of CAD has been based on the evaluation of the anatomic status of epicardial coronary arteries in invasive coronary angiography (ICA). In patients with stable symptoms, however, the diagnostic yield of ICA is low (Patel et al., 2010). Therefore, the current clinical guidelines recommend non‐invasive imaging as the first‐line method in the diagnostic workup of patients with stable symptoms (Gulati et al., 2021; Vrints et al., 2024). These non‐invasive methods include anatomic imaging with coronary computed tomography angiography (CCTA) and functional assessment with myocardial perfusion imaging (MPI).

Among the various MPI modalities, the gold standard is positron emission tomography (PET) using [^15^O]H_2_O. PET MPI with [^15^O]H_2_O enables absolute quantification of myocardial blood flow (MBF) due to the favourable kinetics of the radiotracer (Klein et al., 2020). However, as PET MPI lacks anatomic information, impaired absolute MBF is non‐specific to stenosed epicardial coronary arteries. In addition to flow‐limiting stenoses, MBF impairment may also reflect a disorder in coronary microcirculation (Schindler et al., 2023). Recent studies have suggested that the detection of flow‐limiting stenoses in PET MPI may be improved by evaluating relative perfusion differences across the myocardium (Lopez et al., 2025; Mark et al., 2024). Relative myocardial perfusion impairment is defined as a proportional reduction of regional perfusion in relation to the highest regional perfusion. Still, no prior study has investigated whether these relative differences could be clinically useful in patients with suspected CAD and diffusely impaired myocardial perfusion.

Our goal was to assess whether relative hyperaemic MBF has clinical utility to predict the presence of obstructive CAD in ICA in patients with diffusely impaired myocardial perfusion revealed by [^15^O]H_2_O PET MPI. For this purpose, we utilized a single‐centre registry of patients who underwent CCTA and consecutive [^15^O]H_2_O PET MPI due to suspected obstructive CAD.

## 2 MATERIALS AND METHODS

### 2.1 Patient population

We retrospectively identified all patients referred to CCTA and consecutive [^15^O]H_2_O PET MPI at Kuopio University Hospital, Kuopio, Finland, in 2017–2023. This initial population comprised 971 patients who underwent imaging due to symptoms suggestive of obstructive CAD. The exclusion criteria were technically unreliable CCTA or PET MPI, prior myocardial infarction or coronary revascularization, heart failure, valvular disease of at least moderate degree, cardiomyopathy, and the presence of a pacemaker. After implementing the exclusion criteria, the study population consisted of 874 patients. The final analyses included patients with at least one suspected epicardial stenosis in CCTA, diffusely impaired hyperaemic MBF in PET MPI, and a further referral to ICA. Based on ICA, these patients were then divided into two groups for comparison: patients with non‐obstructive CAD and those with obstructive CAD. Figure 1 presents a flow chart for patient selection.

**FIGURE 1:**
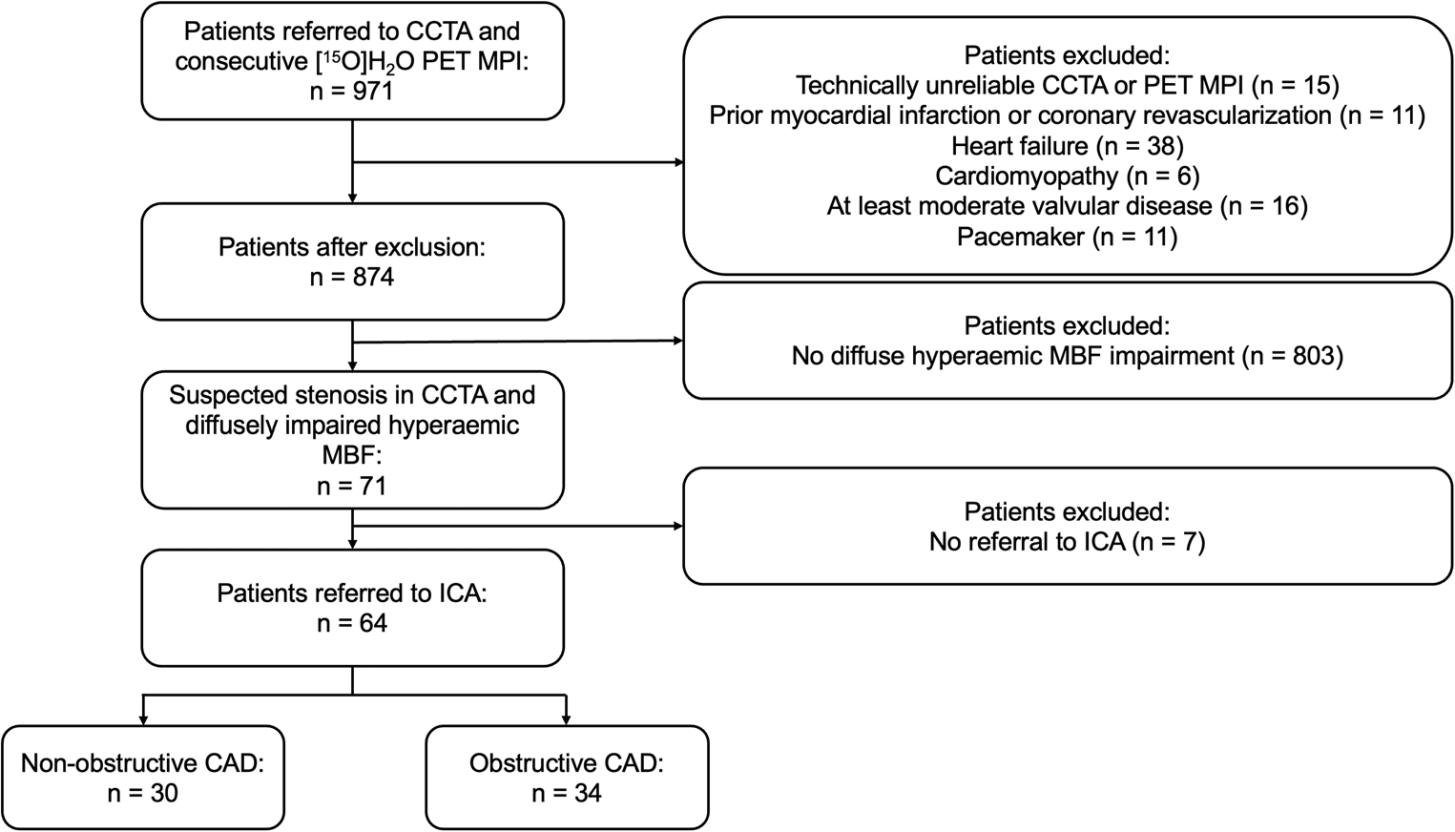
Flow chart for patient selection. CAD, coronary artery disease; CCTA, coronary computed tomography angiography; ICA, invasive coronary angiography; MPI, myocardial perfusion imaging; MBF, myocardial blood flow; PET, positron emission tomography.

The study adhered to the principles of the Declaration of Helsinki. Prior to the study, we acquired written research authorization from the Director of Research and Innovation for the Wellbeing Services County of North Savo (administrative decision § 232/2025). Since the study was retrospective, it required no ethical preapproval or informed patient consent according to the Finnish National Board on Research Integrity.

### 2.2 Clinical data

Clinical data were collected from electronic patient records, and a questionnaire was filled out before PET MPI. The data included age, sex, body mass index, symptoms, risk factors for CAD, and current medication.

### 2.3 Imaging protocol

In accordance with the current practical guidelines, CCTA is the primary imaging method used at Kuopio University Hospital to evaluate CAD in patients with low‐to‐moderate pretest likelihood (Gulati et al., 2021; Vrints et al., 2024). If CCTA is suggestive of a significant stenosis, i.e., if any coronary artery has a luminal stenosis ≥50%, or if significant stenoses cannot be ruled out due to suboptimal image quality, patients undergo consecutive [^15^O]H_2_O PET MPI. Typically, PET MPI is performed on the same day as CCTA. In the case of scheduling issues or inadequate patient preparation, PET MPI is performed on a separate day.

### 2.4 Coronary computed tomography angiography

CCTA was imaged with a Somatom Definition Flash, Somatom Force, or Somatom Edge computed tomography (CT) scanner (Siemens Healthineers, Erlangen, Germany). If the patient's heart rate was over 65 beats per minute, they were given up to 20 mg of intravenous metoprolol. Immediately before imaging, they also received a 1.25 mg dose of sublingual isosorbide dinitrate. The imaging began with a non‐contrast CT used for calcium scoring (Agatston et al., 1990). A consecutive contrast‐enhanced CT was imaged with prospective electrocardiogram‐gating. From the contrast‐enhanced images, we evaluated the left main (LM), left anterior descending (LAD), left circumflex (LCX), and right (RCA) coronary arteries. From each artery, we determined the presence of potentially significant luminal stenoses (≥50%).

### 2.5 Myocardial perfusion imaging

PET MPI was imaged with [^15^O]H_2_O during adenosine‐induced hyperaemia. Imaging was performed with a Biograph mCT or Biograph Vision PET scanner (Siemens Healthineers, Erlangen, Germany). Prior to PET MPI, patients were instructed to abstain from smoking for at least 4 h, caffeine for at least 12 h, and alcohol for at least 48 h. In addition, dipyridamole was discontinued for at least 48 h before PET MPI.

Adenosine was infused at a rate of 140 μg/kg/min for 6.5 min. A bolus of [^15^O]H_2_O with a weight‐adjusted activity of 500–800 MBq was injected 1.5 min after the initiation of adenosine infusion, followed by 5 min of dynamic image acquisition. Attenuation correction was performed with low‐dose CT. An automated sphygmomanometer device (SunTech Tango M2, SunTech Medical Inc., Morrisville, NC, USA) was used to measure blood pressure. Cardiac rhythm and heart rate were monitored with a 12‐lead electrocardiogram using the GE Case (General Electric Company, Boston, MA, USA).

PET images acquired with Biograph mCT were reconstructed with two iterations and 21 subsets, with a 3.18 mm × 3.18 mm pixel size. For images acquired with Biograph Vision, image reconstruction was performed with eight iterations and five subsets, with a pixel size of 2.83 mm × 2.83 mm. A 128 × 128 matrix size and Gaussian 6 mm post‐filtering were used for all images.

Hyperaemic MBF was quantified using Carimas software (Turku PET Centre, Turku, Finland) with the standard 17‐segment model (Cerqueira et al., 2002). Hyperaemic MBF was determined for each individual myocardial segment. In addition, we determined global hyperaemic MBF covering all 17 segments as well as hyperaemic MBF of the standardized coronary territories of LAD, LCX, and RCA. For each patient, the lowest and highest hyperaemic MBFs were determined from the average MBFs between two adjacent myocardial segments. The average hyperaemic MBF between basal septal segments 2 and 3 was excluded. Relative hyperaemic MBF was determined as the percentage ratio of the lowest to the highest hyperaemic MBF. Hyperaemic MBF <2.3 mL/g/min was considered impaired (Danad et al., 2014). Diffusely impaired hyperaemic MBF was defined as hyperaemic MBF <2.3 mL/g/min in all three standardized coronary territories. A repeatability analysis of the MBF quantification is described in the Supplementary Material.

### 2.6 Invasive coronary angiography

Based on the CCTA and PET MPI results, as well as clinical evaluation, the attending cardiologists considered whether to refer a patient to ICA. From ICA, we determined findings from LM, LAD, LCX, and RCA. Obstructive CAD was defined as the presence of luminal stenosis ≥70%, since this degree of stenosis reflects flow‐limiting disease in most patients (Tonino et al., 2010). Conversely, non‐obstructive CAD was defined as the presence of luminal stenosis <70%. Fractional flow reserve (FFR) was measured in only eight patients and thus was not included in the study.

### 2.7 Statistical methods

Statistical analyses were conducted using SPSS version 29 (IBM, Armonk, NY, USA) with statistical significance set at a *p*‐value < 0.05. The normality of distribution of continuous variables was assessed with the Shapiro–Wilk test. Normally distributed continuous variables were presented as mean ± standard deviation or mean (95% confidence interval), whereas non‐normally distributed continuous variables were presented as median (interquartile range). Categorical data were shown as *n* (%). Due to restrictions set by the Finnish Social and Health Data Permit Authority regarding retrospective studies, categorical data with *n* < 3 were prohibited from being presented in exact numbers.

To assess differences between the patient groups in continuous variables, we used Student's *t*‐test for normally distributed data and the Mann–Whitney *U* test for non‐normally distributed data. The presence of between‐group differences in categorical data was tested by either the chi‐squared test or Fisher's exact test, depending on the expected cell count.

The accuracy of relative hyperaemic MBF to detect obstructive CAD was tested with receiver operating characteristic (ROC) curve analysis. Youden's index was used to select the highest discrimination between obstructive and non‐obstructive CAD.

Binary logistic regression analysis was used to assess whether relative hyperaemic MBF was independently associated with the likelihood of obstructive CAD in ICA. Other covariates in the model comprised age, sex, calcium score, and suspected three‐vessel stenosis in CCTA. Statistical significance of the model was evaluated with the Omnibus test. The variables in the model had no significant multicollinearity, that is, the variance inflation factor was consistently less than 2.5 (Dormann et al., 2013; Zuur et al., 2010). When inspecting standardized residuals, we identified no outliers.

## 3 RESULTS

Of the 874 patients included in the study, 71 (8%) had at least one suspected significant epicardial stenosis in CCTA and showed diffusely impaired hyperaemic MBF in PET MPI. Of these patients, 64/71 (90%) were further referred to ICA. The clinical characteristics were similar between the patients referred and those not referred to ICA (Supplementary Table 1). In ICA, obstructive CAD was diagnosed in 34/64 (53%) patients. Non‐obstructive CAD was found in each of the remaining 30/64 (47%) patients, that is, no patient had completely normal epicardial coronary arteries based on ICA. Table 1 presents the clinical characteristics of the 64 patients referred to ICA. Age, the proportion of men, and body mass index were similar between the patient groups. Typical angina was more prevalent in patients with obstructive CAD, whereas atypical angina or non‐anginal chest pain was more common in patients with non‐obstructive CAD.

**TABLE 1:** Clinical characteristics of the patients referred to ICA.

	Non‐obstructive CAD (*n* = 30)	Obstructive CAD (*n* = 34)	*p*‐value
Men	24 (80%)	25 (74%)	0.542
Age (years)	68 ± 8	67 ± 9	0.847
Body mass index (kg/m^2^)	30.2 ± 6.1	30.8 ± 4.9	0.658
Symptom			0.018
Typical angina	<3	12 (35%)	
Atypical angina/non‐anginal chest pain	11 (37%)	6 (18%)	
Dyspnoea	11 (37%)	12 (35%)	
Risk factor			
Hypertension	26 (87%)	30 (88%)	1.000
Hypercholesterolaemia	23 (77%)	31 (91%)	0.169
Diabetes	9 (30%)	11 (32%)	0.839
Smoking history	17 (57%)	18 (53%)	0.511
Family history for CAD	15 (50%)	18 (53%)	0.923
Medication			
ACE inhibitor/AR blocker	21 (70%)	28 (82%)	0.244
Beta blocker	15 (50%)	15 (44%)	0.638
Calcium channel blocker	13 (43%)	13 (38%)	0.679
Diuretic	14 (47%)	12 (35%)	0.355
Long‐acting nitrate	<3	<3	‐
Cholesterol medication	17 (57%)	25 (74%)	0.156
Insulin	6 (20%)	5 (15%)	0.575
Metformin	3 (10%)	11 (32%)	0.031
SGLT2 inhibitor	5 (17%)	5 (15%)	0.829
GLP‐1 RA	<3	3 (9%)	‐

*Note*: Values are either *n* (%) or mean ± standard deviation.

Abbreviations: ACE, angiotensin‐converting enzyme; AR, angiotensin II receptor; CAD, coronary artery disease; GLP‐1 RA, glucagon‐like peptide 1 receptor agonist; ICA, invasive coronary angiography; SGLT2, sodium‐glucose cotransporter 2.

The CCTA and PET MPI results of the patients referred to ICA are shown in Table 2. In CCTA, patients with obstructive CAD had a higher calcium score than those with non‐obstructive CAD did. Also, patients with obstructive CAD had a higher prevalence of suspected three‐vessel stenosis. Heart rate, systolic blood pressure, and diastolic blood pressure were similar between the patient groups both at rest and after the [^15^O]H_2_O bolus. No between‐group differences were found in global hyperaemic MBF or hyperaemic MBF of the standardized coronary territories. However, the lowest hyperaemic MBF and relative hyperaemic MBF were lower in patients with obstructive CAD compared to those with non‐obstructive CAD. Supplementary Table 2 shows the CCTA and PET MPI results of all 71 patients with diffusely impaired hyperaemic MBF. The results demonstrated no difference between patients referred and those not referred to ICA.

**TABLE 2:** CCTA and PET MPI in patients referred to ICA.

	Non‐obstructive CAD (*n* = 30)	Obstructive CAD (*n* = 34)	*p*‐value
CCTA			
Calcium score	512 (134–983)	793 (439–1967)	0.018
Vessels with suspected stenosis			<0.001
Suspected 1‐2‐vessel stenosis	24 (80%)	11 (32%)	
Suspected 3‐vessel stenosis	6 (20%)	23 (68%)	
PET MPI			
HR at rest (beats per minute)	66 (55–74)	64 (57–72)	0.595
Systolic BP at rest (mmHg)	127 ± 19	134 ± 28	0.245
Diastolic BP at rest (mmHg)	70 ± 14	69 ± 10	0.719
HR after [^15^O]H_2_O bolus	74 ± 13	72 ± 12	0.593
Systolic BP after [^15^O]H_2_O bolus	125 ± 20	123 ± 22	0.716
Diastolic BP after [^15^O]H_2_O bolus	65 ± 16	63 ± 10	0.626
Atrial fibrillation during PET MPI	8 (27%)	4 (12%)	0.127
Global hyperaemic MBF (mL/g/min)	1.7 ± 0.4	1.5 ± 0.4	0.101
LAD hyperaemic MBF (mL/g/min)	1.8 (1.5–1.9)	1.6 (1.3–1.9)	0.198
LCX hyperaemic MBF (mL/g/min)	1.7 ± 0.4	1.5 ± 0.5	0.128
RCA hyperaemic MBF (mL/g/min)	1.5 ± 0.4	1.4 ± 0.5	0.091
Lowest hyperaemic MBF (mL/g/min)	1.3 (95% CI 1.2–1.5)	1.0 (95% CI 0.9–1.2)	0.002
Relative hyperaemic MBF (%)	66 (95% CI 62–69)	52 (95% CI 47–57)	<0.001

*Note*: Values are either *n* (%), mean ± standard deviation, mean (95% CI), or median (interquartile range).

Abbreviations: BP, blood pressure; CI, confidence interval; CCTA, coronary computed tomography angiography; CAD, coronary artery disease; HR, heart rate; ICA, invasive coronary angiography; LAD, left anterior descending coronary artery; LCX, left circumflex coronary artery; MPI, myocardial perfusion imaging; MBF, myocardial blood flow; PET, positron emission tomography; RCA, right coronary artery.

In the ROC analysis, the area under the curve was 0.778 (95% confidence interval 0.666–0.891) when relative hyperaemic MBF was used to predict the presence of obstructive CAD (Figure 2). Youden's index was at its highest (0.488) with a relative hyperaemic MBF of 56%. This value predicted obstructive CAD with 59% sensitivity, 90% specificity, 87% positive predictive value, and 66% negative predictive value.

**FIGURE 2:**
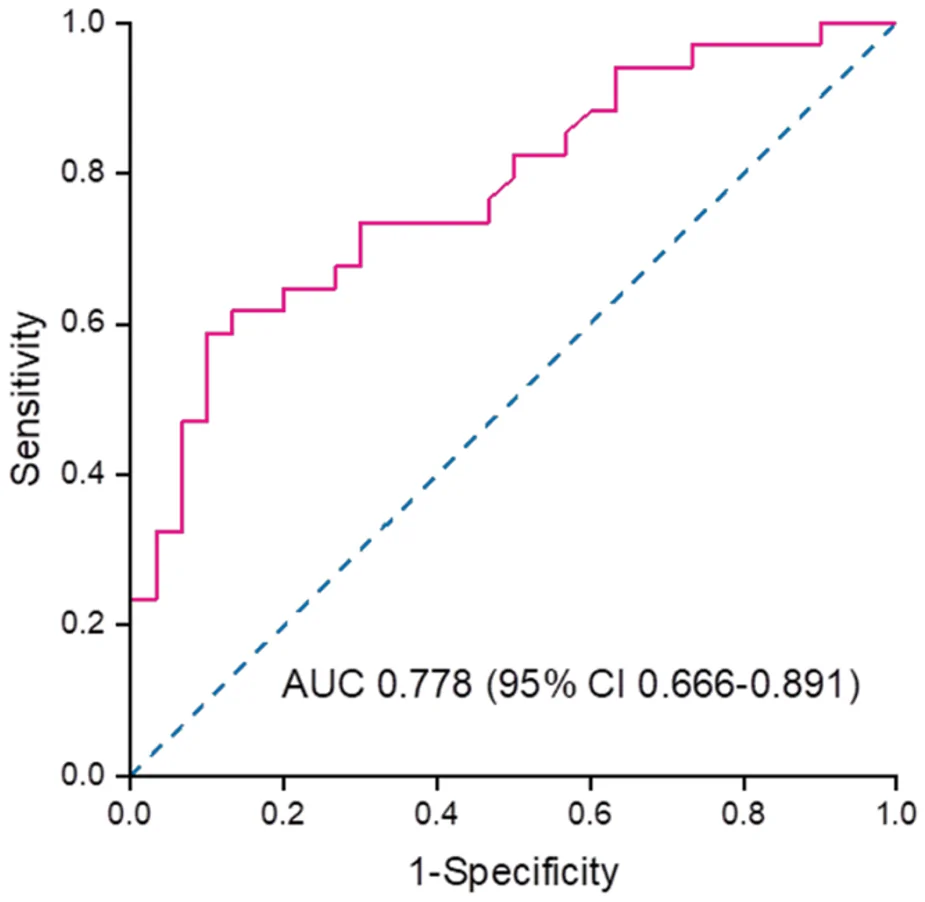
Receiver operating characteristic (ROC) curve analysis of relative myocardial blood flow in the detection of obstructive CAD. AUC, area under the curve; CI, confidence interval.

Finally, Table 3 presents the results of the binary logistic regression analysis. The model was statistically significant (*χ* ^2^ [5, *n* = 64] = 29.38, *p* < 0.001) and correctly classified 81% of the patients. Relative hyperaemic MBF and suspected three‐vessel stenosis in CCTA independently contributed to the model, while age, sex, and calcium score had no independent contribution.

**TABLE 3:** Binary logistic regression predicting obstructive CAD in ICA.

	*β*	*p*‐value	OR	95% CI OR	
				LL	UP
Age	−0.02	0.549	0.98	0.90	1.06
Male sex	−1.04	0.200	0.35	0.07	1.74
Suspected three‐vessel stenosis in CCTA	2.04	0.008	7.71	1.72	34.53
Calcium score	0.00	0.680	1.00	1.00	1.00
Relative hyperaemic MBF	−0.09	0.004	0.92	0.86	0.97
Constant	6.87	0.053	959.65		

Abbreviations: CI, confidence interval; CCTA, coronary computed tomography angiography; LL, lower limit; MBF, myocardial blood flow; OR, odds ratio; UL, upper limit.

## 4 DISCUSSION

Our study showed that among 874 patients undergoing CCTA and PET MPI due to symptoms suggestive of CAD, 71 (8%) had both a suspected epicardial coronary artery stenosis in CCTA and diffusely impaired myocardial perfusion based on [^15^O]H_2_O PET MPI. Of the 64/71 patients referred to ICA, however, obstructive CAD was present in only 34 (53%). Compared to the 30 patients with non‐obstructive CAD in ICA, those with obstructive CAD had more heterogenous myocardial perfusion, as measured with relative hyperaemic MBF.

Only few studies have investigated the prevalence of diffusely impaired myocardial perfusion in patients imaged with consecutive CCTA and PET MPI. Maaniitty et al. reported that in symptomatic patients undergoing CCTA and PET MPI, diffusely impaired myocardial perfusion was present in 16% (Maaniitty et al., 2021). A study by Stenström et al. from the same centre showed that in patients with intermediate pretest likelihood for obstructive CAD, this prevalence was 17% (Stenström et al., 2017). Although diffusely impaired myocardial perfusion was defined similarly in these two studies, the reported prevalences were higher than that in our study. Compared to the exclusion criteria used by Maaniitty et al., those in our study were somewhat stricter, since we excluded patients with at least moderate valvular disease and those with a pacemaker. Stenström et al. specifically studied patients with intermediate pretest likelihood for obstructive CAD, whereas our study population also included patients with a lower pretest likelihood. Nevertheless, based on the previous two studies and our results, the prevalence of diffusely impaired myocardial perfusion is 8–17% in symptomatic patients undergoing CCTA and PET MPI.

Previous studies have shown that impairment in absolute hyperaemic MBF most reliably detects flow‐limiting stenosis in epicardial coronary arteries and is superior to relative measurements of MBF (Danad et al., 2014; Kajander et al., 2011). Still, relative hyperaemic MBF may have additional value to detect obstructive CAD. Relative hyperaemic MBF—defined as the proportional reduction of MBF in relation to the MBF in a reference region—could non‐invasively reflect FFR (De Bruyne et al., 1994). Recently, Mark et al. showed that in a heterogenous patient population, relative hyperaemic MBF in [^15^O]H_2_O PET MPI was independently associated with obstructive CAD (Mark et al., 2024). Further, Lopez et al. studied the utility of relative flow reserve with ^18^F‐flurpiridaz PET MPI (Lopez et al., 2025). They defined relative flow reserve as the ratio of the lowest segmental MBF to the MBF in the coronary territory with the highest MBF. In per‐vessel analysis, relative flow reserve was significantly lower in obstructive CAD (≥70% stenosis in ICA) compared to non‐obstructive CAD (0.55 vs. 0.80). These results align with our per‐patient results. To our knowledge, no previous study has evaluated the clinical utility of relative MBF to detect obstructive CAD specifically in patients with diffusely impaired hyperaemic MBF. Therefore, our results expand existing knowledge by showing that in this patient group, lower relative hyperaemic MBF was independently associated with obstructive CAD. Conversely, in patients with non‐obstructive CAD, the distribution of hyperaemic MBF was more homogenous.

Diffusely impaired hyperaemic MBF is a clinically relevant finding. In comparison with focal impairment, diffusely impaired hyperaemic MBF is a stronger predictor of mortality (Moody et al., 2023). However, diffusely impaired hyperaemic MBF constitutes a diagnostic problem. First, in a large clinical cohort, diffusely impaired hyperaemic MBF was paradoxically associated with a lower likelihood of obstructive CAD (Poitrasson‐Rivière et al., 2023). Second, high plaque burden, that is, diffuse atherosclerosis, is associated with impaired hyperaemic MBF even in the absence of significant stenoses (Maltseva et al., 2025; Schuijf et al., 2020; Vattay et al., 2022). Third, diffusely impaired MBF may indicate coronary microvascular dysfunction (CMD) (Schindler et al., 2023). CMD is a disorder caused by functional or structural pathological processes in the coronary microcirculation, leading to myocardial ischaemia even in the absence of obstructive epicardial CAD (Del Buono et al., 2021). CMD is assumed to be a diffuse process, and indeed, post hoc analysis of the PACIFIC trials showed that CMD was homogeneously distributed across all coronary territories (Hoshino et al., 2025). PET MPI assesses myocardial perfusion of the entire coronary artery tree and cannot differentiate between flow limitation in the epicardial arteries and in the microcirculation. In addition to absolute measurements of MBF, relative hyperaemic MBF could provide a clinically useful perfusion marker for clarifying the distribution of regional flow limitations and eventually the underlying flow‐limiting pathology. Accordingly, our ROC analysis showed that relative hyperaemic MBF ≤56%, had a high specificity (90%) and positive predictive value (87%) for diagnosing obstructive CAD. This indicates that in patients with relative hyperaemic MBF ≤56%, obstructive CAD can be ruled in with high confidence. On the other hand, due to lower sensitivity (59%) and negative predictive value (66%), a relative hyperaemic MBF >56% is unreliable for ruling out obstructive CAD.

In our study, CCTA and ICA results were somewhat discrepant. Each patient had at least one suspected significant stenosis in CCTA, whereas only 53% of them were diagnosed with obstructive CAD in ICA. This is partly due to the different cut‐off values for significant stenosis used in CCTA and ICA. In accordance with the latest practical guideline, PET MPI was performed when ≥50% stenosis was observed in CCTA (Cury et al., 2022). In ICA, however, obstructive CAD was defined as the presence of luminal stenosis ≥70%, which more likely reflects flow‐limiting disease (Tonino et al., 2010). Furthermore, when ICA is used as the reference standard, CCTA has lower specificity for detecting obstructive lesions, resulting in a higher false positive rate (Nielsen et al., 2014). Discrepancies between CCTA and ICA have been reported even when using a 320‐slice high‐performance CT scanner (Song et al., 2019). One explanation for false positives in CCTA is the presence of calcified plaques, which lead to an overestimation of stenosis severity due to blooming artifact (Kalisz et al., 2016; Yan et al., 2013). The patients in our study were imaged with conventional CT scanners. Fortunately, CT technology has significantly improved with the introduction of photon‐counting CT scanners (Willemink et al., 2018). In a recent study, CCTA performed with a photon‐counting CT scanner had excellent diagnostic accuracy (Sakai et al., 2025). However, in present‐day clinical practice, the availability of photon‐counting CT scanners is still limited, and therefore, high calcium burden complicates CCTA interpretation. Thus, in patients with multivessel calcified plaques, relative hyperaemic MBF can help in differentiating significant stenotic lesions from non‐significant ones.

The presence of obstructive CAD in ICA was assessed from the degree of luminal stenosis, and FFR was performed in only a few patients. Still, the significance of routinely performed FFR is questionable. In the pioneering FAME study, FFR‐guided percutaneous coronary intervention of patients with multivessel CAD improved the prognosis at 1 year (Tonino et al., 2009). In contrast, the FUTURE trial found no evidence that FFR‐guided treatment would lead to a better 1‐year prognosis in patients with multivessel CAD (Rioufol et al., 2021). Moreover, several meta‐analyses have shown conflicting results regarding the prognostic significance of FFR‐guided treatment strategy (Elbadawi et al., 2022; Liu et al., 2025; Maznyczka et al., 2022; Yin et al., 2025). Thus, while the use of FFR might have been optimal from a physiological standpoint, visual assessment of stenoses is still both clinically and scientifically valuable, making our results justifiable.

We acknowledge that this single‐centre study has limitations. Although the initial dataset was large (almost 1000 patients), the number of patients remaining in the final analyses was limited. As a result, the statistical power was compromised, increasing the risk for a type 2 error. However, the analyzed dataset was still powerful enough to demonstrate the potential clinical utility of relative perfusion. In addition, the proportion of women was significantly lower than that of men, which may affect the generalizability of our results. As this study was retrospective, some potentially useful data were lacking. For example, as our standard clinical protocol includes only hyperaemic imaging, resting myocardial perfusion images were unavailable for most patients. Resting MBF and myocardial flow reserve were thus not included in this study. The decision to refer a patient to ICA was left to the attending cardiologists, which may have induced some referral bias. This, in turn, may have influenced the estimates of diagnostic performance. However, our research protocol and patient population correspond to clinical practice and current practical guidelines, and therefore, the results of our study can be applied to real‐life patients.

## 5 CONCLUSION

In patients with suspected significant stenosis in CCTA and diffusely impaired myocardial perfusion based on [^15^O]H_2_O PET MPI, relative hyperaemic MBF may have clinical utility for predicting obstructive CAD. The presence of obstructive CAD in ICA is more likely when a patient has lower relative—that is, more heterogenous—hyperaemic MBF. Conversely, diffusely impaired but more homogeneously distributed hyperaemic MBF is predictive of non‐obstructive CAD and may relate to CMD.

## CONFLICT OF INTEREST STATEMENT

The authors declare no conflicts of interest.

## ETHICS STATEMENT

The Director of Research and Innovation for the Wellbeing Services County of North Savo issued an administrative decision (§ 232/2025) granting research authorization for this study.

## Supporting information

Supporting File 1.

## Data Availability

The data underlying this manuscript cannot be publicly shared since it contains sensitive health information and data access is subject to statutory restrictions and the terms of the relevant data permits. The researchers are not authorized to share individual‐level data to third parties. Eligible researchers may apply for data access from the Wellbeing Services County of North Savo (email: kirjaamo@pshyvinvointialue.fi). All applications will be independently evaluated by the data controllers and the data permit authority.
