# Supplementary material for: Diffusely impaired myocardial perfusion in patients with suspected coronary artery disease: Clinical utility of relative perfusion
Source: Clin Physiol Funct Imaging. 2026 Sep 29;46(6):e70096. doi: 10.1111/cpf.70096 (PMC13624522; doi:10.1111/cpf.70096)
Supplement: Supplementary file 1 [file CPF-46-0-s001.docx]

**Supplementary Material**

**Repeatability of myocardial blood flow quantification**

We evaluated the repeatability of MBF quantification by reprocessing the same [^15^O]H₂O PET MPI dataset under both intra-observer and inter-observer settings. For intra-observer repeatability, the intraclass correlation coefficient (ICC) was 0.983 for global hyperaemic MBF, and 0.982, 0.980, and 0.976 for hyperaemic MBF in the LAD, LCX, and RCA territories, respectively. The corresponding coefficients of variation (CV%) were 4.5% for global hyperaemic MBF and 4.7%, 4.6%, and 5.6% for hyperaemic MBF in the LAD, LCX, and RCA territories, respectively. For inter-observer repeatability, the ICC was 0.966 for global hyperaemic MBF, and 0.972, 0.967, and 0.968 for hyperaemic MBF in the LAD, LCX, and RCA territories, respectively. The corresponding CV% values were 6.0% for global hyperaemic MBF and 5.7%, 5.6%, and 6.3% for hyperaemic MBF in the LAD, LCX, and RCA territories, respectively.

**Supplementary Table 1** Clinical characteristics of the patients with suspected stenosis in CCTA and diffusely impaired hyperaemic MBF

|  | No ICA  (n = 7) | ICA  (n = 64) | *p*-value |
| --- | --- | --- | --- |
| Men | 6 (86%) | 49 (77%) | 1.000 |
| Age (years) | 71 ± 8 | 67 ± 8 | 0.298 |
| Body mass index | 29.7 ± 7.7 | 30.5 ± 5.5 | 0.730 |
|  |  |  |  |
| Symptom |  |  |  |
| Typical angina | 3 (43%) | NA* | - |
| Atypical angina/nonanginal chest pain | < 3 | 17 (27%) | - |
| Dyspnoea | < 3 | 23 (36%) | - |
|  |  |  |  |
| Risk factor |  |  |  |
| Hypertension | 5 (71%) | 56 (88%) | 0.577 |
| Hypercholesterolaemia | 6 (86%) | 54 (84%) | 0.583 |
| Diabetes | < 3 | 20 (31%) | - |
| Smoking history | < 3 | 35 (55%) | - |
| Family history for CAD | 6 (86%) | 33 (52%) | 0.123 |
|  |  |  |  |
| Medication |  |  |  |
| ACE inhibitor/AR blocker | 4 (57%) | 49 (77%) | 0.628 |
| Beta blocker | 3 (43%) | 30 (47%) | 1.000 |
| Calcium channel blocker | < 3 | 26 (41%) | - |
| Diuretic | < 3 | 26 (41%) | - |
| Long-acting nitrate | < 3 | NA* | - |
| Cholesterol medication | 6 (86%) | 42 (66%) | 0.167 |
| Insulin | 0 | 11 (17%) | 0.580 |
| Metformin | < 3 | 14 (22%) | - |
| SGLT2 inhibitor | 0 | 10 (16%) | 0.583 |
| GLP-1 RA | 0 | NA* | - |

Values are either n (%) or mean ± standard deviation. ICA = invasive coronary angiography, NA = not available, CAD = coronary artery disease, ACE = angiotensin-converting enzyme, AR = angiotensin II receptor, SGLT2 = sodium-glucose cotransporter 2, GLP-1 RA = glucagon-like peptide 1 receptor agonist.

* = result not available due to restrictions set by the Finnish Social and Health Data Permit Authority

**Supplementary Table 2** CCTA and PET MPI results of the patients with suspected stenosis in CCTA and diffusely impaired hyperaemic MBF

|  | No ICA  (n = 7) | ICA  (n = 64) | *p*-value |
| --- | --- | --- | --- |
| CCTA |  |  |  |
| Calcium score | 509 (43–1276) | 661 (266–1262) | 0.407 |
| Vessels with suspected stenosis |  |  |  |
| Suspected 1–2-vessel stenosis | NA* | 35 (55%) | - |
| Suspected 3-vessel stenosis | NA* | 29 (45%) | - |
|  |  |  |  |
| PET MPI |  |  |  |
| HR at rest (beats per minute) | 58 (50–61) | 64 (56–72) | 0.071 |
| Systolic BP at rest (mmHg) | 130 (100–162) | 130 (114–140) | 0.992 |
| Diastolic BP at rest (mmHg) | 67 (44–81) | 69 (61–76) | 0.481 |
| HR after [^15^O]H_2_O bolus | 65 ± 11 | 73 ± 13 | 0.135 |
| Systolic BP after [^15^O]H_2_O bolus | 128 ± 25 | 124 ± 21 | 0.645 |
| Diastolic BP after [^15^O]H_2_O bolus | 66 (55-84) | 65 (56-71) | 0.563 |
| Atrial fibrillation during PET MPI | < 3 | 12 (19%) | - |
| Global hyperaemic MBF (mL/g/min) | 1.6 (1.0–1.7) | 1.7 (1.3–1.9) | 0.310 |
| LAD hyperaemic MBF (mL/g/min) | 1.5 (1.0–1.6) | 1.7 (1.3–1.9) | 0.231 |
| LCX hyperaemic MBF (mL/g/min) | 1.7 (1.0–1.8) | 1.7 (1.3–2.0) | 0.629 |
| Lowest hyperaemic MBF (mL/g/min) | 1.1 ± 0.5 | 1.2 ± 0.4 | 0.678 |
| Relative hyperaemic MBF (%) | 62 ± 10 | 59 ± 14 | 0.590 |

Values are either n (%), mean ± standard deviation, or median (interquartile range). ICA = invasive coronary angiography, CCTA = coronary computed tomography angiography, NA = not available, PET = positron emission tomography, MPI = myocardial perfusion imaging, CAD = coronary artery disease, HR = heart rate, BP = blood pressure, CI = confidence interval, MBF = myocardial blood flow, LAD = left anterior descending coronary artery, LCX = left circumflex coronary artery, RCA = right coronary artery.

* = result not available due to restrictions set by the Finnish Social and Health Data Permit Authority
